# Surgical resection of a tuberculoma in the diaphragm: a case report

**DOI:** 10.1186/s40792-022-01554-y

**Published:** 2022-10-14

**Authors:** Asato Hashinokuchi, Mikihiro Kohno, Keisuke Kosai, Yuki Ono, Naoki Haratake, Daiki Shibata, Hidetaka Yamamoto, Tomoyoshi Takenaka, Tomoharu Yoshizumi

**Affiliations:** 1 Department of Surgery and Science, Graduate School of Medical Sciences, Kyushu University, 3-1-1, Maidashi, Higashi-ku, Fukuoka, 812-8582 Japan; 2 Department of Anatomic Pathology and Pathological Sciences, Graduate School of Medical Sciences, Kyushu University, 3-1-1, Maidashi, Higashi-ku, Fukuoka, 812-8582 Japan

**Keywords:** Tuberculoma, Diaphragm, Extrapulmonary tuberculosis, Paradoxical reaction

## Abstract

**Background:**

Extrapulmonary tuberculosis commonly affects the lymphatic system, nervous system, and gastrointestinal system. Tuberculous infection in the muscle is very rare. Moreover, tuberculous infection in the diaphragm is extremely rare. We herein report a case of tuberculomas in the diaphragm and posterior mediastinum that were successfully diagnosed and treated.

**Case presentation:**

We encountered a 62-year-old woman with a tuberculoma in the diaphragm. The patient presented with mild dyspnea. Computed tomography showed a mass in the left diaphragm, focal thickening of the posterior mediastinum, and multiple nodules in the lungs. Positron emission tomography–computed tomography showed increased uptake in the left diaphragm mass and thickening of the posterior mediastinum; therefore, we considered the masses to be malignant and planned surgical resection. However, the patient was diagnosed with tuberculosis from a sputum culture, and she was treated with anti-tuberculous therapy. The masses in the diaphragm and posterior mediastinum had become enlarged after 6 months of anti-tuberculous therapy; therefore, the patient underwent resection of both masses. Tuberculous infection was histologically confirmed in each lesion. She was pathologically diagnosed with tuberculous abscesses in the diaphragm and posterior mediastinum and began treatment with anti-tuberculosis drugs.

**Conclusions:**

Preoperative diagnosis of a tuberculoma in the diaphragm is usually difficult, and surgical intervention is important for both diagnosis and treatment.

## Background

Tuberculosis is still a major health concern, although the economic status of the general population and the efficacy of anti-tuberculosis drugs have been improved. The lung is one of the most commonly infected organs in patients with tuberculosis, and extrapulmonary tuberculosis commonly affects the lymphatic system, nervous system, and gastrointestinal system [1]. Tuberculous infection in the muscle is extremely rare, occurring in about 0.015% of all patients with tuberculosis [2]. Moreover, a few cases of tuberculous infection in the diaphragm have been reported [2–4]. We herein report a case of tuberculomas in the diaphragm and posterior mediastinum that were successfully diagnosed and treated.

## Case presentation

**Fig. 1:**
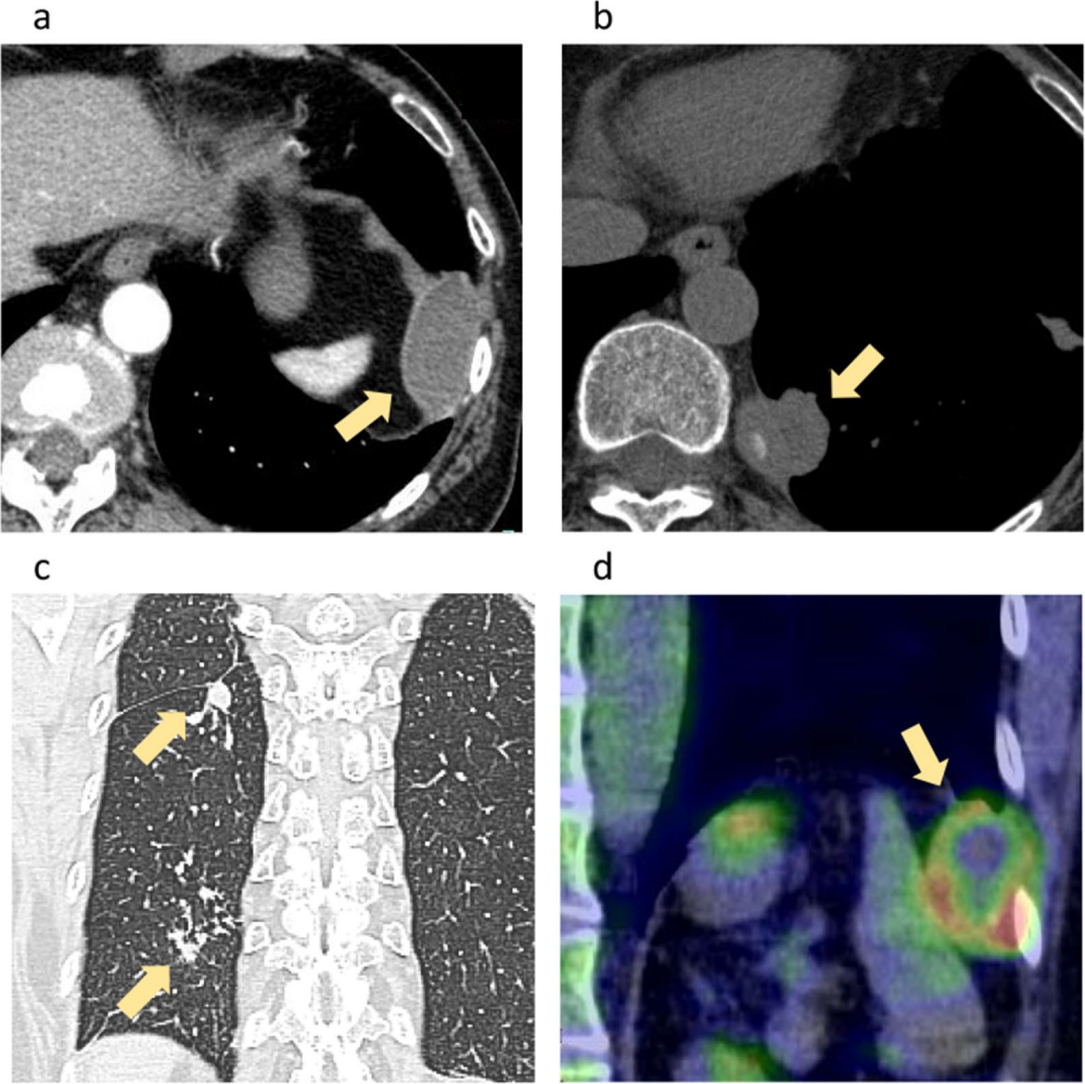
CT and PET–CT. High-resolution lung CT images showed (**a**) a cystic lesion in the left diaphragm, **b** focal thickening of the posterior mediastinal pleura with calcification, and **c** multiple small nodules in both lung fields. **d** PET–CT (coronal view) showed increased uptake of fluorodeoxyglucose in the cystic lesion. CT, computed tomography; PET–CT, positron emission tomography–computed tomography

This case involved a 62-year-old woman with tuberculomas in the diaphragm and posterior mediastinum. She had no relevant medical history and had never smoked. She presented with mild dyspnea. Chest X-ray examination showed small nodules in the bilateral lung fields. Blood test results were normal, including the tumor markers carcinoembryonic antigen and carbohydrate antigen 19–9. Computed tomography (CT) of the chest showed a cystic lesion measuring 30 mm in the left diaphragm (Fig. 1a), focal thickening of the posterior mediastinal pleura with calcification in the region of the left 11th thoracic vertebra (Fig. 1b), and multiple small nodules in the bilateral lungs (Fig. 1c). Positron emission tomography–CT revealed increased uptake of fluorodeoxyglucose in the cystic lesion in the left diaphragm [maximum standardized uptake value (SUV_max_) = 6.9] (Fig. 1d) and posterior mediastinum (SUV_max_ = 2.1). Surgical resection of the masses in the left diaphragm and posterior mediastinum was considered because of concern regarding malignancy. However, a sputum culture showed *Mycobacterium tuberculosis*; therefore, the patient was treated with anti-tuberculosis therapy (ATT) before surgery. A sputum culture was negative 6 months later. Although treatment for tuberculosis seemed successful, the diaphragmatic mass slightly increased in size and the posterior mediastinal thickness became considerably enlarged. We thus considered that these lesions could be malignant, and the patient was admitted to our hospital for surgery. We did not perform a percutaneous CT-guided needle biopsy of diaphragmatic mass for the diagnosis, because we concerned that a percutaneous biopsy might cause a dissemination of the tuberculosis or the tumor.

**Fig. 2:**
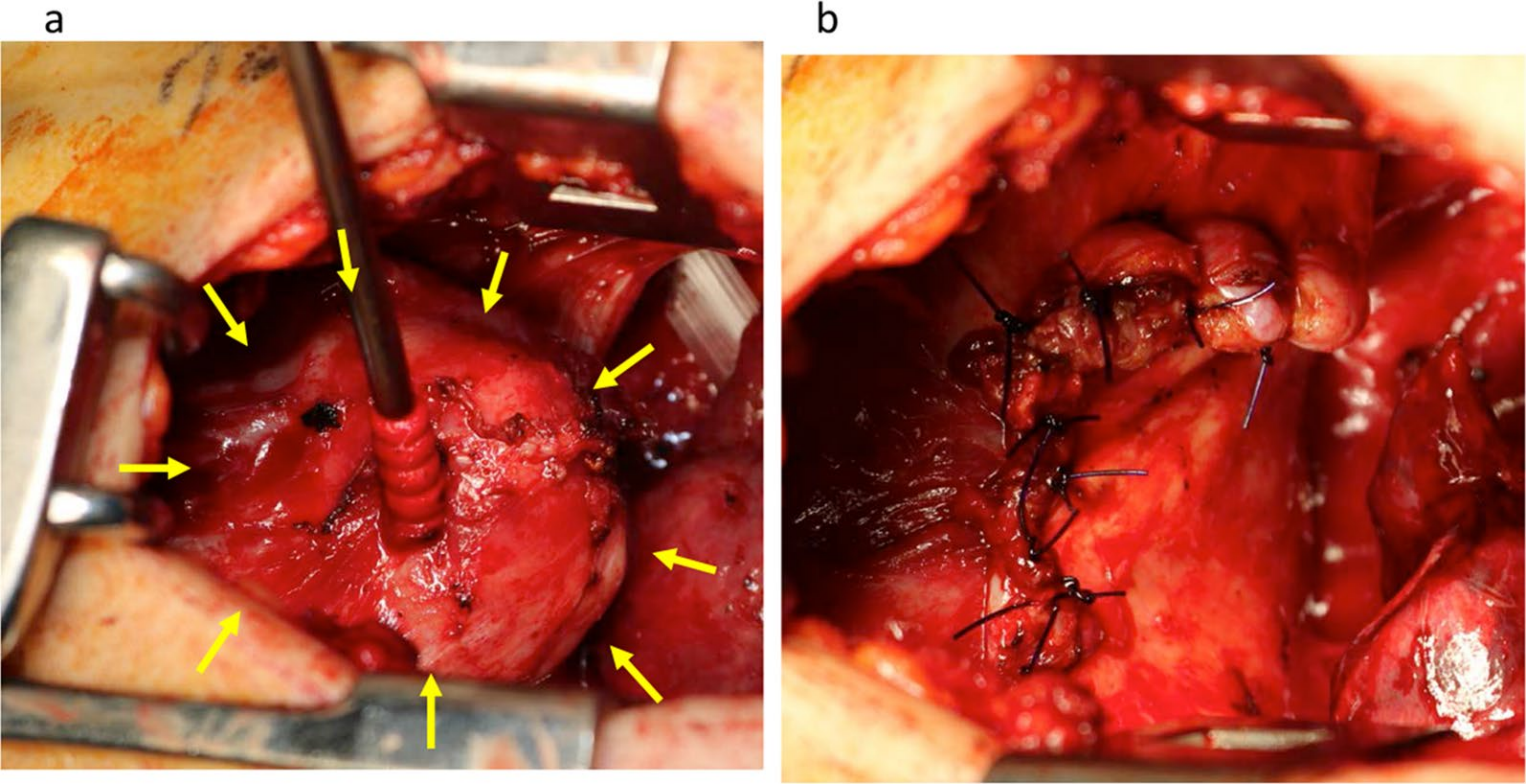
Intraoperative findings. **a** 68-mm mass was present in the diaphragm. **b** We resected the mass with a healthy diaphragm margin and reconstructed the diaphragm by simple suturing

After the patient and her family had provided informed consent for surgery, we performed the operation. Because we considered the possibility of the tuberculoma in the diaphragm, we performed the surgery in the negative pressure operating room. We placed an 11.5-mm port on the anterior axillary line of the sixth intercostal space, but we found a dense inflammatory adhesion between the lung and chest wall. Therefore, the chest cavity was opened through the eighth intercostal space. We detected the mass in the diaphragm (Fig. 2a) and posterior mediastinum. A dense adhesion was also present between the mass in the diaphragm and left lower lobe, and we performed wedge resection of the left lower lobe, because it was adhered to the mass. We then divided the diaphragm, leaving a healthy muscle margin around the lesion, and reconstructed the diaphragm with simple suturing (Fig. 2b). There was no adhesion between the left diaphragm and the abdominal organs. We also resected the posterior mediastinal lesion with wedge resection of the left lower lobe. The diaphragmatic lesion was filled with purulent liquid.

Polymerase chain reaction of the purulent liquid in the left diaphragm was positive for tuberculosis, and the lesion was, therefore, diagnosed as a tuberculoma. Acid-fast bacteria culture was negative. The patient had an uncomplicated postoperative course and began treatment with anti-tuberculosis drugs comprising isoniazid (300 mg/day), rifampicin (450 mg/day), ethambutol (750 mg/day), and pyrazinamide (1200 mg/day) on postoperative day 7. She was discharged on postoperative day 10 with no complications. At the 6-month follow-up, she was asymptomatic with no evidence of recurrence on CT.

**Fig. 3:**
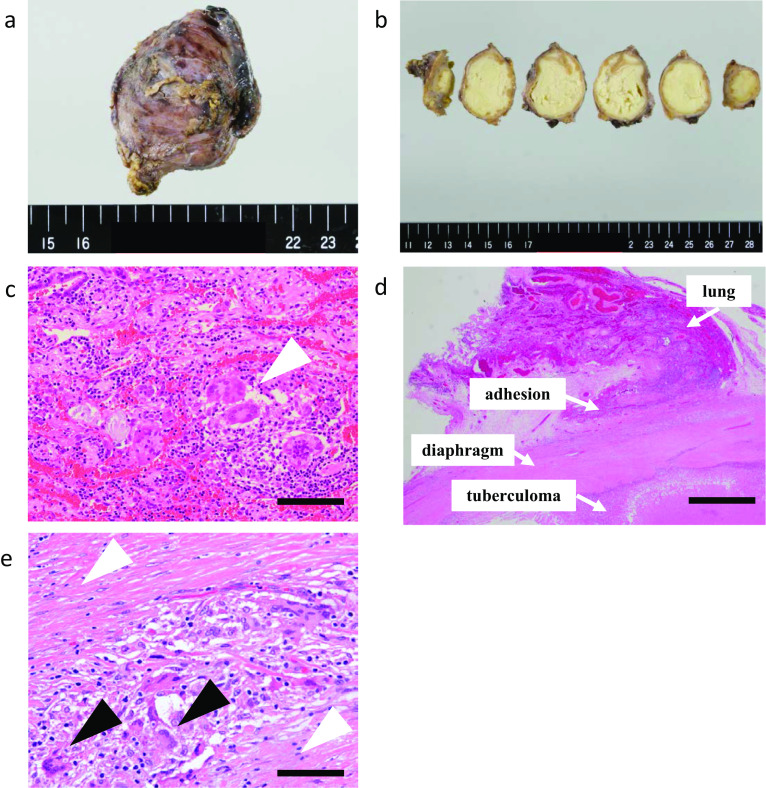
Macroscopic findings and histopathological findings. **a**, **b** Mass in the left diaphragm was 68 mm in size, and the cut surface showed yellow content covered with a thick wall. **c** Conventional hematoxylin- and eosin-stained section of lung tissue adhered to the tuberculoma showed epithelioid cell granulomas composed of histiocytes and multinucleated giant cells (white arrow heads). Bar: 100 µm. **d** Adhesion was present between the tuberculoma and left lower lung. Bar: 1 mm. **e** Histiocytes and multinucleated giant cells (black arrow heads) were found in the diaphragmatic muscular layer (white arrow heads). Bar: 50 µm

Macroscopic examination of the lesion in the left diaphragm showed a 68-mm yellow mass surrounded by a thick wall (Fig. 3a, b). Microscopic examination of both specimens showed epithelioid cell granulomas composed of histiocytes and multinucleated giant cells with caseous necrosis (Fig. 3c, d), although no acid-fast bacilli were detected by Ziehl–Neelsen staining. The tuberculoma was surrounded by diaphragmatic muscle tissue (Fig. 3d). In addition, there were histiocytes and multinucleated giant cells in the diaphragmatic muscular layer (Fig. 3e). Therefore, the patient was pathologically diagnosed with tuberculous abscesses in the diaphragm and posterior mediastinum, and additional ATT was administered.

## Discussion

We have herein reported a rare case of a tuberculoma in the diaphragm. Extrapulmonary tuberculosis in the muscles is very rare, with an incidence rate of 0.015% [2], and only a few cases of extrapulmonary tuberculosis in the diaphragm have been published (Table 1) [2–4]. Cold abscesses are defined abscesses that lack acute inflammation signs, such as pain, erythema, heat, swelling and immobility [5]. Cold abscesses usually result from tuberculous infection [5]. In the present case, the patient did not show any chest pain, heat or erythema. The diaphragmatic tuberculoma was considered as a cold abscess.

**Table 1:** Reported cases of patients with diaphragmatic tuberculoma

Case report (References)	Age (years)	Symptoms	History	Preoperative diagnosis	Diagnosis	Preoperative ATT	Postoperative ATT	CT findings		
								Pulmonary lesions		Enhancement of abscess wall
Tharun et al., 2018 [2]	38	Right upper abdominal pain	N.A.	−	Surgery	+	+	N.A		N.A
Peng et al., 2016 [3]	22	Night sweats, right upper abdominal pain	−	−	Surgery	−	+	−		+
Inoue et al., 2010 [4]	84	−	−	−	Surgery	−	+	−		+
Present case	62	Dyspnea	−	−	Surgery	+	+	+		+

*N.A.* not available, *ATT* anti-tuberculous therapy, *CT* computed tomography

Including the present case, four cases involving treatment of a tuberculoma in the diaphragm have been reported [2–4]. The patients required surgical resection in all cases, although ATT was preoperatively performed in two cases. In addition, surgical treatment should be considered for treatment of all diaphragmatic tumors, because there is always a possibility of malignancy [4]. In our case, the mass in the diaphragm enlarged despite 6 months of ATT, and we performed surgical resection, because we considered the possibility of malignancy. Therefore, surgical treatment should be considered for tuberculomas in the diaphragm.

Extrapulmonary tuberculosis can be challenging to diagnose on the basis of imaging features [1]. Enhancement of the abscess wall on CT images was seen in three patients, making it difficult to differentiate between an abscess and a malignancy. Diaphragmatic tumors are difficult to diagnose by noninvasive approaches, such as CT and ultrasonography [4]. In addition, two previously described patients had no tuberculous lesions in the bilateral lung fields; thus, a definitive diagnosis of a tuberculous abscess was difficult to attain preoperatively [3, 4]. In some previous studies, the diagnostic yield for tuberculosis was 85–95% when the biopsy was performed by video-assisted surgery for tuberculous peritonitis [6]. Therefore, surgical intervention may be useful not only for treatment but also for diagnosis.

A paradoxical reaction, which is defined as a transient clinical or radiological worsening of pre-existing tuberculous lesions or the development of new lesions in patients undergoing ATT, may also make the diagnosis difficult [7]. A paradoxical reaction is seen in 2% of patients with tuberculous, and its incidence is certainly higher in patients with extrapulmonary tuberculosis, such as that characterized by lesions in the lymph nodes, central nervous system, and respiratory system [8]. The causes of paradoxical reactions are not clear, but such reactions may occur as a result of recovery of the immune system with an enhanced inflammatory reaction [9]. In the present case, pre-existing tuberculous lesions in the diaphragm and posterior mediastinum worsened after ATT; therefore, we considered the possibility of a paradoxical reaction. However, we performed surgical resection to attain a diagnosis, because it is difficult to distinguish worsening by paradoxical reaction from proliferation of malignancy.

Possible mechanisms underlying the development of tuberculosis in the chest wall include spread via the lymphatics, spread via the bloodstream, and dissemination from the lung to the thoracic space [10]. Peng et al. [3] suggested that hematogenous dissemination was the route of spread leading to a tuberculous abscess in the diaphragm. In our patient, pathologic examination revealed lung tissue with multinucleated giant cells adhered to the wall of the tuberculosis lesions in both the diaphragm and posterior mediastinum (Fig. 3c, e). There were many multinucleated giant cells not only in the lung tissue and the tuberculoma but also in the diaphragmatic muscular layer between the adhered lung tissue and tuberculoma. Therefore, the pathogenesis of a tuberculoma in the diaphragm could involve direct invasion of the diaphragm by the pulmonary tuberculosis, resulting in a diaphragmatic abscess.

## Conclusions

This report has presented a rare case of a tuberculoma in the diaphragm. The diagnosis of a tuberculoma in the diaphragm is difficult without resection, because symptoms and imaging features of tuberculosis are variable and the development of a paradoxical reaction makes it difficult to assess the worsening of tuberculous lesions. Moreover, conservative treatment is mostly ineffective for tuberculomas in the diaphragm. Therefore, surgical resection of tuberculomas in the diaphragm may be important for both diagnosis and treatment.

## Data Availability

Not applicable.
